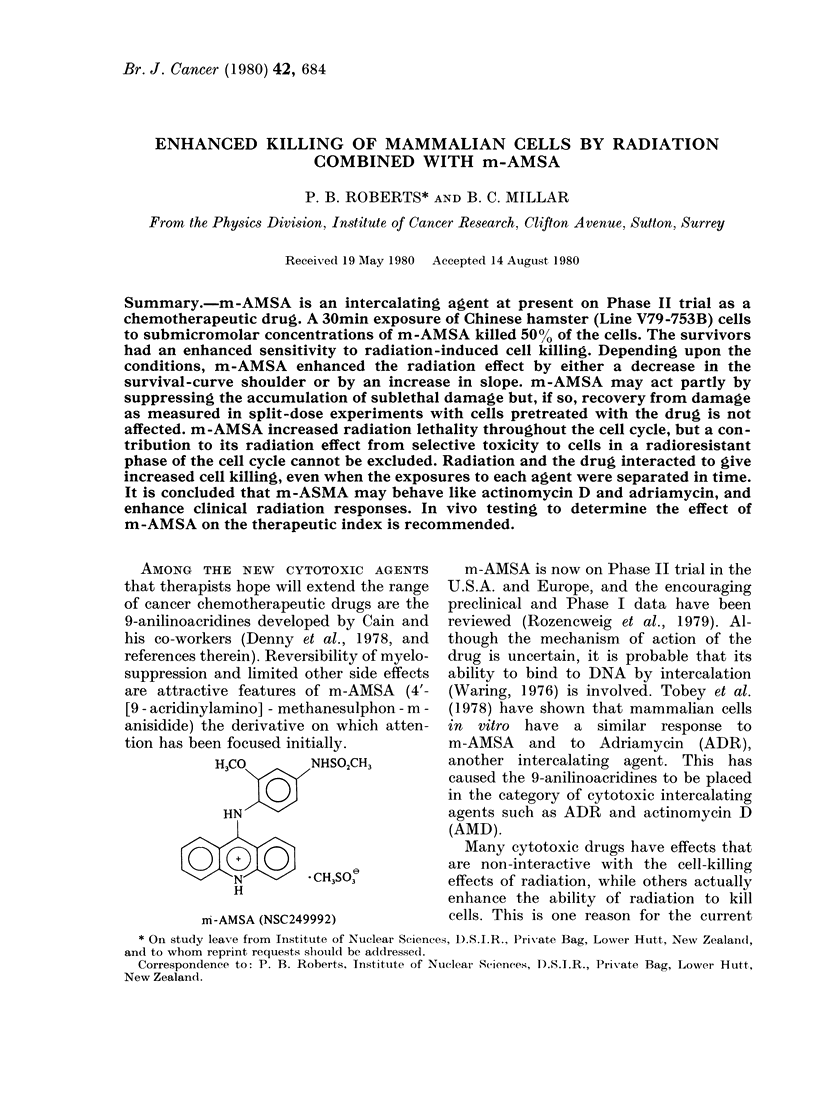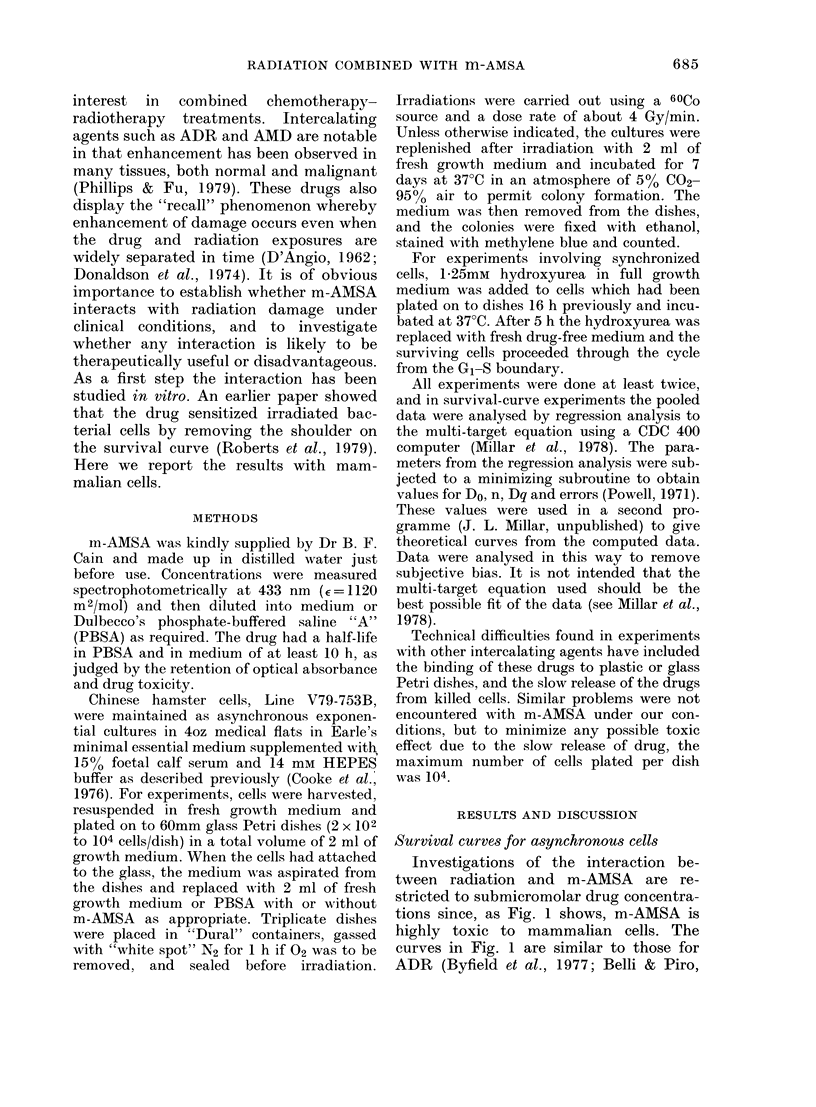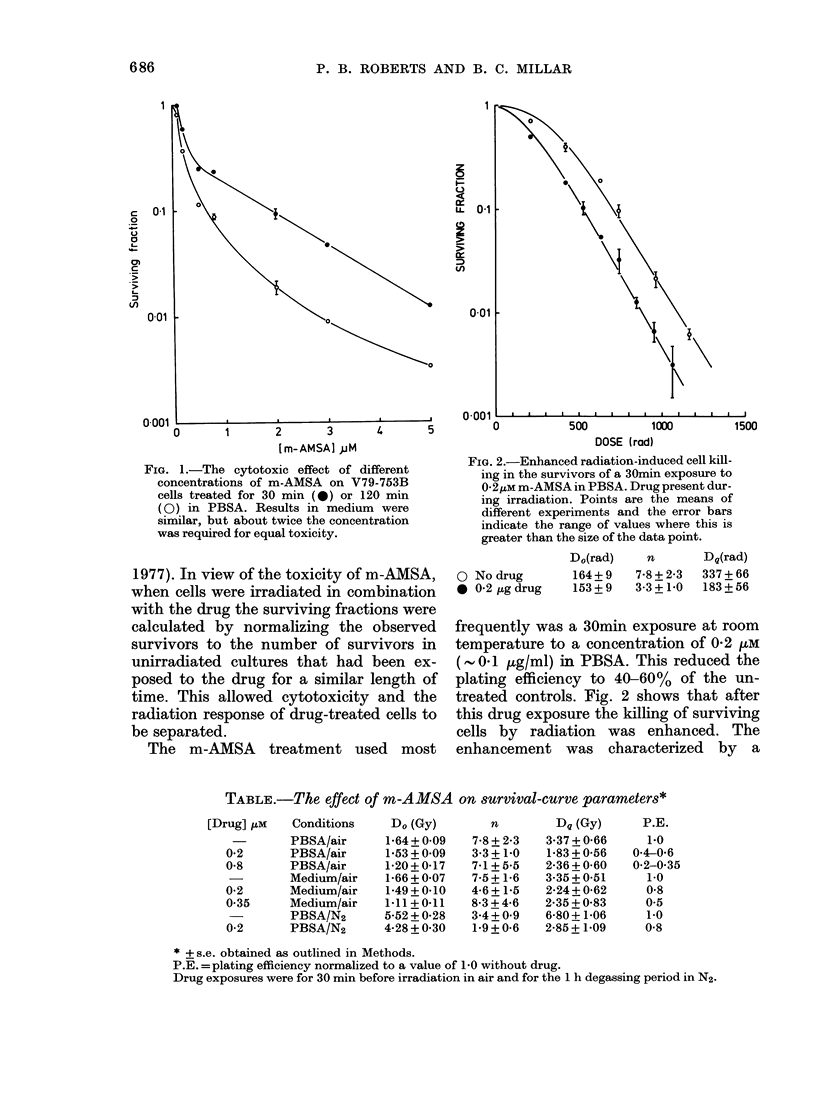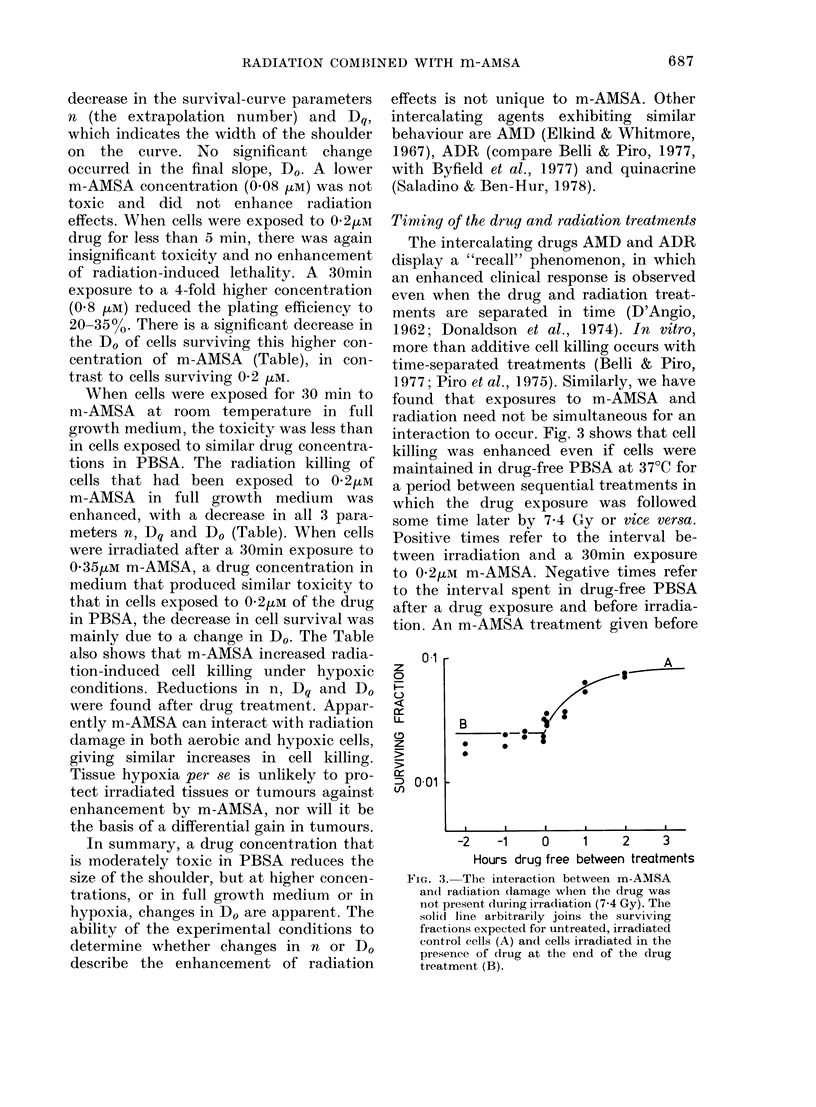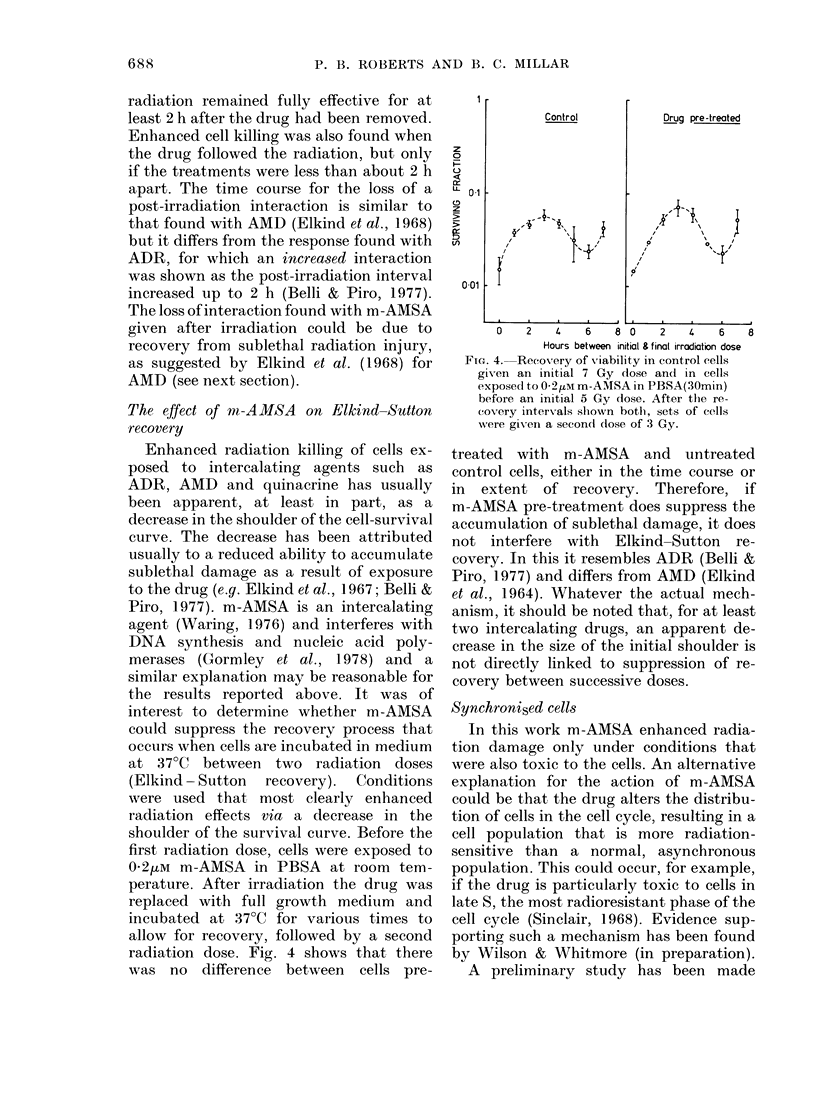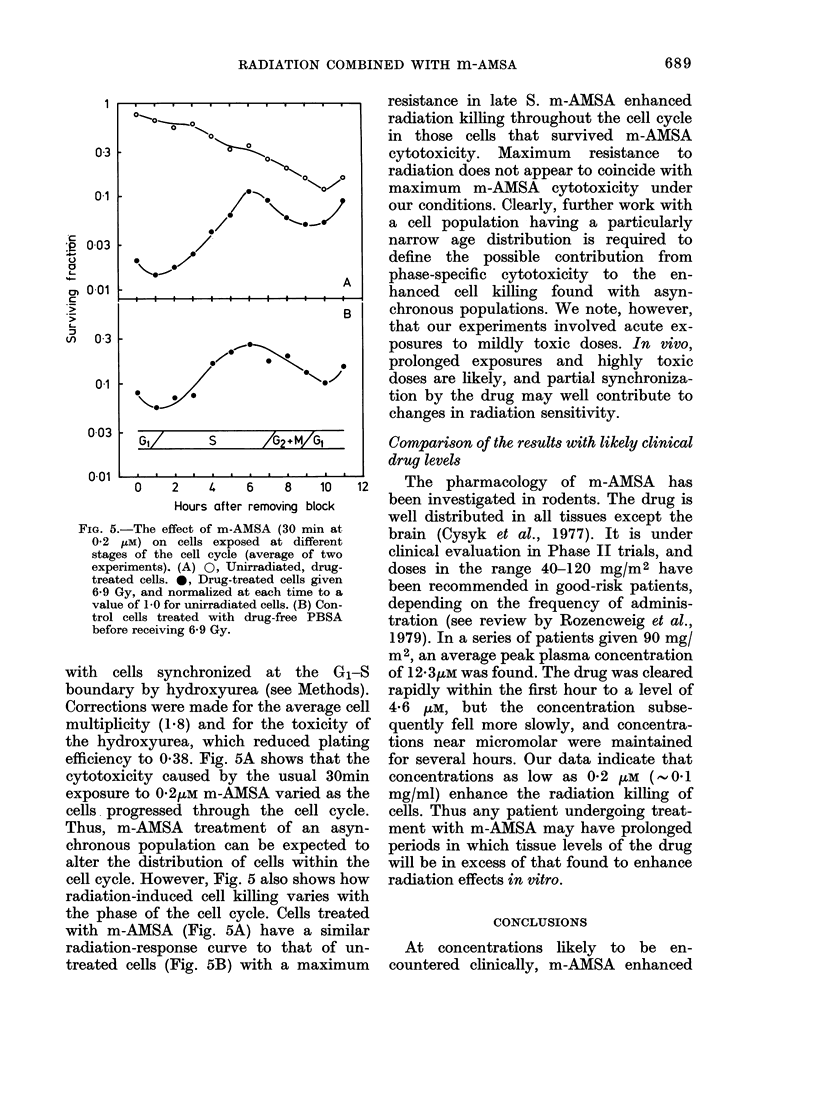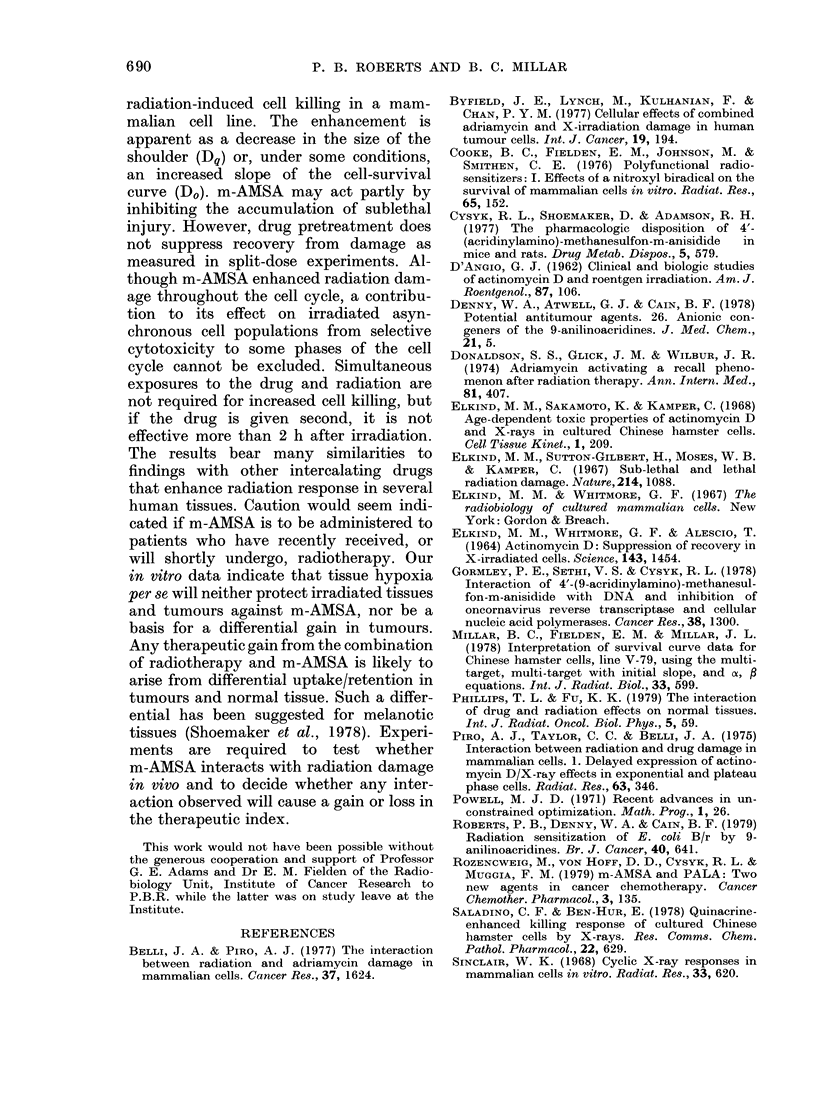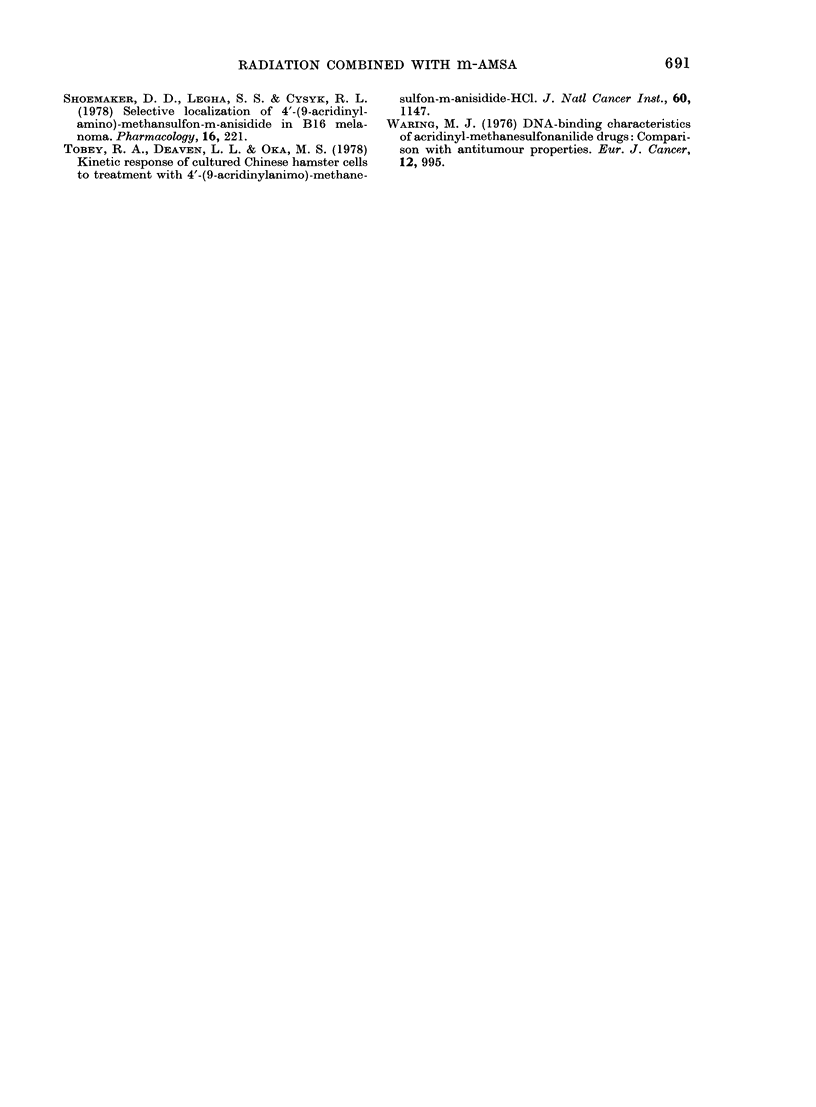# Enhanced killing of mammalian cells by radiation combined with m-AMSA.

**DOI:** 10.1038/bjc.1980.302

**Published:** 1980-11

**Authors:** P. B. Roberts, B. C. Millar

## Abstract

m-AMSA is an intercalating agent at present on Phase II trial as a chemotherapeutic drug. A 30 min exposure of Chinese hamster (Line V79-753B) cells to submicromolar concentrations of m-AMSA killed 50% of the cells. The survivors had an enhanced sensitivity to radiation-induced cell killing. Depending upon the conditions, m-AMSA enhanced the radiation effect by either a decrease in the survival-curve shoulder or by an increase in slope. m-AMSA may act partly by suppressing the accumulation of sublethal damage but, if so, recovery from damage as measured in split-dose experiments with cells pretreated with the drug is not affected to its radiation effect from selective toxicity to cells in a radioresistant phase of the cell cycle cannot be excluded. Radiation and the drug interacted to give increased cell killing, even when the exposures to each agent were separated in time. It is concluded that m-ASMA may behave like actinomycin D and adriamycin, and enhance clinical radiation responses. In vivo testing to determine the effect of m-AMSA on the therapeutic index is recommended.


*Br. J. Cancer* (1980) **42**, 684

### ENHANCED KILLING OF MAMMALIAN CELLS BY RADIATION COMBINED WITH m-AMSA

P. B. ROBERTS* AND B. C. MILLAR

*From the Physics Division, Institute of Cancer Research, Clifton Avenue, Sutton, Surrey*

Received 19 May 1980 Accepted 14 August 1980

**Summary.—m-AMSA is an intercalating agent at present on Phase II trial as a chemotherapeutic drug. A 30min exposure of Chinese hamster (Line V79-753B) cells to submicromolar concentrations of m-AMSA killed 50% of the cells. The survivors had an enhanced sensitivity to radiation-induced cell killing. Depending upon the conditions, m-AMSA enhanced the radiation effect by either a decrease in the survival-curve shoulder or by an increase in slope. m-AMSA may act partly by suppressing the accumulation of sublethal damage but, if so, recovery from damage as measured in split-dose experiments with cells pretreated with the drug is not affected. m-AMSA increased radiation lethality throughout the cell cycle, but a contribution to its radiation effect from selective toxicity to cells in a radioresistant phase of the cell cycle cannot be excluded. Radiation and the drug interacted to give increased cell killing, even when the exposures to each agent were separated in time. It is concluded that m-ASMA may behave like actinomycin D and adriamycin, and enhance clinical radiation responses. In vivo testing to determine the effect of m-AMSA on the therapeutic index is recommended.**

AMONG THE NEW CYTOTOXIC AGENTS that therapists hope will extend the range of cancer chemotherapeutic drugs are the 9-anilinoacridines developed by Cain and his co-workers (Denny *et al.*, 1978, and references therein). Reversibility of myelosuppression and limited other side effects are attractive features of m-AMSA (4'-[9-acridinylamino]-methanesulphon-m-anisidide) the derivative on which attention has been focused initially.

m-AMSA (NSC249992)

m-AMSA is now on Phase II trial in the U.S.A. and Europe, and the encouraging preclinical and Phase I data have been reviewed (Rozencweig *et al.*, 1979). Although the mechanism of action of the drug is uncertain, it is probable that its ability to bind to DNA by intercalation (Waring, 1976) is involved. Tobey *et al.* (1978) have shown that mammalian cells *in vitro* have a similar response to m-AMSA and to Adriamycin (ADR), another intercalating agent. This has caused the 9-anilinoacridines to be placed in the category of cytotoxic intercalating agents such as ADR and actinomycin D (AMD).

Many cytotoxic drugs have effects that are non-interactive with the cell-killing effects of radiation, while others actually enhance the ability of radiation to kill cells. This is one reason for the current

* On study leave from Institute of Nuclear Sciences, D.S.I.R., Private Bag, Lower Hutt, New Zealand, and to whom reprint requests should be addressed.

Correspondence to: P. B. Roberts, Institute of Nuclear Sciences, D.S.I.R., Private Bag, Lower Hutt, New Zealand.

interest in combined chemotherapy–radiotherapy treatments. Intercalating agents such as ADR and AMD are notable in that enhancement has been observed in many tissues, both normal and malignant (Phillips & Fu, 1979). These drugs also display the "recall" phenomenon whereby enhancement of damage occurs even when the drug and radiation exposures are widely separated in time (D'Angio, 1962; Donaldson *et al.*, 1974). It is of obvious importance to establish whether m-AMSA interacts with radiation damage under clinical conditions, and to investigate whether any interaction is likely to be therapeutically useful or disadvantageous. As a first step the interaction has been studied *in vitro*. An earlier paper showed that the drug sensitized irradiated bacterial cells by removing the shoulder on the survival curve (Roberts *et al.*, 1979). Here we report the results with mammalian cells.

### METHODS

m-AMSA was kindly supplied by Dr B. F. Cain and made up in distilled water just before use. Concentrations were measured spectrophotometrically at 433 nm ($\epsilon = 1120$ $m^2$/mol) and then diluted into medium or Dulbecco's phosphate-buffered saline "A" (PBSA) as required. The drug had a half-life in PBSA and in medium of at least 10 h, as judged by the retention of optical absorbance and drug toxicity.

Chinese hamster cells, Line V79-753B, were maintained as asynchronous exponential cultures in 4oz medical flats in Earle's minimal essential medium supplemented with 15% foetal calf serum and 14 mM HEPES buffer as described previously (Cooke *et al.*, 1976). For experiments, cells were harvested, resuspended in fresh growth medium and plated on to 60mm glass Petri dishes ($2 \times 10^2$ to $10^4$ cells/dish) in a total volume of 2 ml of growth medium. When the cells had attached to the glass, the medium was aspirated from the dishes and replaced with 2 ml of fresh growth medium or PBSA with or without m-AMSA as appropriate. Triplicate dishes were placed in "Dural" containers, gassed with "white spot" $N_2$ for 1 h if $O_2$ was to be removed, and sealed before irradiation. Irradiations were carried out using a $^{60}Co$ source and a dose rate of about 4 Gy/min. Unless otherwise indicated, the cultures were replenished after irradiation with 2 ml of fresh growth medium and incubated for 7 days at 37°C in an atmosphere of 5% $CO_2$–95% air to permit colony formation. The medium was then removed from the dishes, and the colonies were fixed with ethanol, stained with methylene blue and counted.

For experiments involving synchronized cells, 1·25mM hydroxyurea in full growth medium was added to cells which had been plated on to dishes 16 h previously and incubated at 37°C. After 5 h the hydroxyurea was replaced with fresh drug-free medium and the surviving cells proceeded through the cycle from the $G_1$–S boundary.

All experiments were done at least twice, and in survival-curve experiments the pooled data were analysed by regression analysis to the multi-target equation using a CDC 400 computer (Millar *et al.*, 1978). The parameters from the regression analysis were subjected to a minimizing subroutine to obtain values for $D_0$, n, D*q* and errors (Powell, 1971). These values were used in a second programme (J. L. Millar, unpublished) to give theoretical curves from the computed data. Data were analysed in this way to remove subjective bias. It is not intended that the multi-target equation used should be the best possible fit of the data (see Millar *et al.*, 1978).

Technical difficulties found in experiments with other intercalating agents have included the binding of these drugs to plastic or glass Petri dishes, and the slow release of the drugs from killed cells. Similar problems were not encountered with m-AMSA under our conditions, but to minimize any possible toxic effect due to the slow release of drug, the maximum number of cells plated per dish was $10^4$.

### RESULTS AND DISCUSSION

### *Survival curves for asynchronous cells*

Investigations of the interaction between radiation and m-AMSA are restricted to submicromolar drug concentrations since, as Fig. 1 shows, m-AMSA is highly toxic to mammalian cells. The curves in Fig. 1 are similar to those for ADR (Byfield *et al.*, 1977; Belli & Piro,

FIG. 1.—The cytotoxic effect of different concentrations of m-AMSA on V79-753B cells treated for 30 min (●) or 120 min (○) in PBSA. Results in medium were similar, but about twice the concentration was required for equal toxicity.

FIG. 2.—Enhanced radiation-induced cell killing in the survivors of a 30min exposure to 0·2μM m-AMSA in PBSA. Drug present during irradiation. Points are the means of different experiments and the error bars indicate the range of values where this is greater than the size of the data point.

| | $D_0$(rad) | *n* | $D_q$(rad) |
|---|---|---|---|
| ○ No drug | 164 ± 9 | 7·8 ± 2·3 | 337 ± 66 |
| ● 0·2 μg drug | 153 ± 9 | 3·3 ± 1·0 | 183 ± 56 |

1977). In view of the toxicity of m-AMSA, when cells were irradiated in combination with the drug the surviving fractions were calculated by normalizing the observed survivors to the number of survivors in unirradiated cultures that had been exposed to the drug for a similar length of time. This allowed cytotoxicity and the radiation response of drug-treated cells to be separated.

The m-AMSA treatment used most frequently was a 30min exposure at room temperature to a concentration of 0·2 μM (~0·1 μg/ml) in PBSA. This reduced the plating efficiency to 40–60% of the untreated controls. Fig. 2 shows that after this drug exposure the killing of surviving cells by radiation was enhanced. The enhancement was characterized by a

TABLE.—*The effect of m-AMSA on survival-curve parameters**

| [Drug] μM | Conditions | $D_0$ (Gy) | *n* | $D_q$ (Gy) | P.E. |
|---|---|---|---|---|---|
| — | PBSA/air | 1·64 ± 0·09 | 7·8 ± 2·3 | 3·37 ± 0·66 | 1·0 |
| 0·2 | PBSA/air | 1·53 ± 0·09 | 3·3 ± 1·0 | 1·83 ± 0·56 | 0·4–0·6 |
| 0·8 | PBSA/air | 1·20 ± 0·17 | 7·1 ± 5·5 | 2·36 ± 0·60 | 0·2–0·35 |
| — | Medium/air | 1·66 ± 0·07 | 7·5 ± 1·6 | 3·35 ± 0·51 | 1·0 |
| 0·2 | Medium/air | 1·49 ± 0·10 | 4·6 ± 1·5 | 2·24 ± 0·62 | 0·8 |
| 0·35 | Medium/air | 1·11 ± 0·11 | 8·3 ± 4·6 | 2·35 ± 0·83 | 0·5 |
| — | PBSA/$N_2$ | 5·52 ± 0·28 | 3·4 ± 0·9 | 6·80 ± 1·06 | 1·0 |
| 0·2 | PBSA/$N_2$ | 4·28 ± 0·30 | 1·9 ± 0·6 | 2·85 ± 1·09 | 0·8 |

* ± s.e. obtained as outlined in Methods.
P.E. = plating efficiency normalized to a value of 1·0 without drug.
Drug exposures were for 30 min before irradiation in air and for the 1 h degassing period in $N_2$.

decrease in the survival-curve parameters $n$ (the extrapolation number) and $D_q$, which indicates the width of the shoulder on the curve. No significant change occurred in the final slope, $D_0$. A lower m-AMSA concentration (0·08 $\mu$M) was not toxic and did not enhance radiation effects. When cells were exposed to 0·2$\mu$M drug for less than 5 min, there was again insignificant toxicity and no enhancement of radiation-induced lethality. A 30min exposure to a 4-fold higher concentration (0·8 $\mu$M) reduced the plating efficiency to 20–35%. There is a significant decrease in the $D_0$ of cells surviving this higher concentration of m-AMSA (Table), in contrast to cells surviving 0·2 $\mu$M.

When cells were exposed for 30 min to m-AMSA at room temperature in full growth medium, the toxicity was less than in cells exposed to similar drug concentrations in PBSA. The radiation killing of cells that had been exposed to 0·2$\mu$M m-AMSA in full growth medium was enhanced, with a decrease in all 3 parameters $n$, $D_q$ and $D_0$ (Table). When cells were irradiated after a 30min exposure to 0·35$\mu$M m-AMSA, a drug concentration in medium that produced similar toxicity to that in cells exposed to 0·2$\mu$M of the drug in PBSA, the decrease in cell survival was mainly due to a change in $D_0$. The Table also shows that m-AMSA increased radiation-induced cell killing under hypoxic conditions. Reductions in n, $D_q$ and $D_0$ were found after drug treatment. Apparently m-AMSA can interact with radiation damage in both aerobic and hypoxic cells, giving similar increases in cell killing. Tissue hypoxia *per se* is unlikely to protect irradiated tissues or tumours against enhancement by m-AMSA, nor will it be the basis of a differential gain in tumours.

In summary, a drug concentration that is moderately toxic in PBSA reduces the size of the shoulder, but at higher concentrations, or in full growth medium or in hypoxia, changes in $D_0$ are apparent. The ability of the experimental conditions to determine whether changes in $n$ or $D_0$ describe the enhancement of radiation effects is not unique to m-AMSA. Other intercalating agents exhibiting similar behaviour are AMD (Elkind & Whitmore, 1967), ADR (compare Belli & Piro, 1977, with Byfield *et al.*, 1977) and quinacrine (Saladino & Ben-Hur, 1978).

*Timing of the drug and radiation treatments*

The intercalating drugs AMD and ADR display a "recall" phenomenon, in which an enhanced clinical response is observed even when the drug and radiation treatments are separated in time (D'Angio, 1962; Donaldson *et al.*, 1974). *In vitro*, more than additive cell killing occurs with time-separated treatments (Belli & Piro, 1977; Piro *et al.*, 1975). Similarly, we have found that exposures to m-AMSA and radiation need not be simultaneous for an interaction to occur. Fig. 3 shows that cell killing was enhanced even if cells were maintained in drug-free PBSA at 37°C for a period between sequential treatments in which the drug exposure was followed some time later by 7·4 Gy or *vice versa.* Positive times refer to the interval between irradiation and a 30min exposure to 0·2$\mu$M m-AMSA. Negative times refer to the interval spent in drug-free PBSA after a drug exposure and before irradiation. An m-AMSA treatment given before

FIG. 3.—The interaction between m-AMSA and radiation damage when the drug was not present during irradiation (7·4 Gy). The solid line arbitrarily joins the surviving fractions expected for untreated, irradiated control cells (A) and cells irradiated in the presence of drug at the end of the drug treatment (B).

radiation remained fully effective for at least 2 h after the drug had been removed. Enhanced cell killing was also found when the drug followed the radiation, but only if the treatments were less than about 2 h apart. The time course for the loss of a post-irradiation interaction is similar to that found with AMD (Elkind *et al.*, 1968) but it differs from the response found with ADR, for which an *increased* interaction was shown as the post-irradiation interval increased up to 2 h (Belli & Piro, 1977). The loss of interaction found with m-AMSA given after irradiation could be due to recovery from sublethal radiation injury, as suggested by Elkind *et al.* (1968) for AMD (see next section).

*The effect of m-AMSA on Elkind–Sutton recovery*

Enhanced radiation killing of cells exposed to intercalating agents such as ADR, AMD and quinacrine has usually been apparent, at least in part, as a decrease in the shoulder of the cell-survival curve. The decrease has been attributed usually to a reduced ability to accumulate sublethal damage as a result of exposure to the drug (*e.g.* Elkind *et al.*, 1967; Belli & Piro, 1977). m-AMSA is an intercalating agent (Waring, 1976) and interferes with DNA synthesis and nucleic acid polymerases (Gormley *et al.*, 1978) and a similar explanation may be reasonable for the results reported above. It was of interest to determine whether m-AMSA could suppress the recovery process that occurs when cells are incubated in medium at 37°C between two radiation doses (Elkind - Sutton recovery). Conditions were used that most clearly enhanced radiation effects *via* a decrease in the shoulder of the survival curve. Before the first radiation dose, cells were exposed to 0·2$\mu$M m-AMSA in PBSA at room temperature. After irradiation the drug was replaced with full growth medium and incubated at 37°C for various times to allow for recovery, followed by a second radiation dose. Fig. 4 shows that there was no difference between cells pre-

FIG. 4.—Recovery of viability in control cells given an initial 7 Gy dose and in cells exposed to 0·2$\mu$M m-AMSA in PBSA(30min) before an initial 5 Gy dose. After the recovery intervals shown both, sets of cells were given a second dose of 3 Gy.

treated with m-AMSA and untreated control cells, either in the time course or in extent of recovery. Therefore, if m-AMSA pre-treatment does suppress the accumulation of sublethal damage, it does not interfere with Elkind–Sutton recovery. In this it resembles ADR (Belli & Piro, 1977) and differs from AMD (Elkind *et al.*, 1964). Whatever the actual mechanism, it should be noted that, for at least two intercalating drugs, an apparent decrease in the size of the initial shoulder is not directly linked to suppression of recovery between successive doses.

*Synchronised cells*

In this work m-AMSA enhanced radiation damage only under conditions that were also toxic to the cells. An alternative explanation for the action of m-AMSA could be that the drug alters the distribution of cells in the cell cycle, resulting in a cell population that is more radiation-sensitive than a normal, asynchronous population. This could occur, for example, if the drug is particularly toxic to cells in late S, the most radioresistant phase of the cell cycle (Sinclair, 1968). Evidence supporting such a mechanism has been found by Wilson & Whitmore (in preparation).

A preliminary study has been made

FIG. 5.—The effect of m-AMSA (30 min at 0·2 μM) on cells exposed at different stages of the cell cycle (average of two experiments). (A) ○, Unirradiated, drug-treated cells. ●, Drug-treated cells given 6·9 Gy, and normalized at each time to a value of 1·0 for unirradiated cells. (B) Control cells treated with drug-free PBSA before receiving 6·9 Gy.

with cells synchronized at the $G_1$–S boundary by hydroxyurea (see Methods). Corrections were made for the average cell multiplicity (1·8) and for the toxicity of the hydroxyurea, which reduced plating efficiency to 0·38. Fig. 5A shows that the cytotoxicity caused by the usual 30min exposure to 0·2μM m-AMSA varied as the cells progressed through the cell cycle. Thus, m-AMSA treatment of an asynchronous population can be expected to alter the distribution of cells within the cell cycle. However, Fig. 5 also shows how radiation-induced cell killing varies with the phase of the cell cycle. Cells treated with m-AMSA (Fig. 5A) have a similar radiation-response curve to that of untreated cells (Fig. 5B) with a maximum resistance in late S. m-AMSA enhanced radiation killing throughout the cell cycle in those cells that survived m-AMSA cytotoxicity. Maximum resistance to radiation does not appear to coincide with maximum m-AMSA cytotoxicity under our conditions. Clearly, further work with a cell population having a particularly narrow age distribution is required to define the possible contribution from phase-specific cytotoxicity to the enhanced cell killing found with asynchronous populations. We note, however, that our experiments involved acute exposures to mildly toxic doses. *In vivo,* prolonged exposures and highly toxic doses are likely, and partial synchronization by the drug may well contribute to changes in radiation sensitivity.

### *Comparison of the results with likely clinical drug levels*

The pharmacology of m-AMSA has been investigated in rodents. The drug is well distributed in all tissues except the brain (Cysyk *et al.*, 1977). It is under clinical evaluation in Phase II trials, and doses in the range 40–120 mg/m$^2$ have been recommended in good-risk patients, depending on the frequency of administration (see review by Rozencweig *et al.*, 1979). In a series of patients given 90 mg/m$^2$, an average peak plasma concentration of 12·3μM was found. The drug was cleared rapidly within the first hour to a level of 4·6 μM, but the concentration subsequently fell more slowly, and concentrations near micromolar were maintained for several hours. Our data indicate that concentrations as low as 0·2 μM (~0·1 mg/ml) enhance the radiation killing of cells. Thus any patient undergoing treatment with m-AMSA may have prolonged periods in which tissue levels of the drug will be in excess of that found to enhance radiation effects *in vitro.*

### CONCLUSIONS

At concentrations likely to be encountered clinically, m-AMSA enhanced radiation-induced cell killing in a mammalian cell line. The enhancement is apparent as a decrease in the size of the shoulder ($D_q$) or, under some conditions, an increased slope of the cell-survival curve ($D_o$). m-AMSA may act partly by inhibiting the accumulation of sublethal injury. However, drug pretreatment does not suppress recovery from damage as measured in split-dose experiments. Although m-AMSA enhanced radiation damage throughout the cell cycle, a contribution to its effect on irradiated asynchronous cell populations from selective cytotoxicity to some phases of the cell cycle cannot be excluded. Simultaneous exposures to the drug and radiation are not required for increased cell killing, but if the drug is given second, it is not effective more than 2 h after irradiation. The results bear many similarities to findings with other intercalating drugs that enhance radiation response in several human tissues. Caution would seem indicated if m-AMSA is to be administered to patients who have recently received, or will shortly undergo, radiotherapy. Our *in vitro* data indicate that tissue hypoxia *per se* will neither protect irradiated tissues and tumours against m-AMSA, nor be a basis for a differential gain in tumours. Any therapeutic gain from the combination of radiotherapy and m-AMSA is likely to arise from differential uptake/retention in tumours and normal tissue. Such a differential has been suggested for melanotic tissues (Shoemaker *et al.*, 1978). Experiments are required to test whether m-AMSA interacts with radiation damage *in vivo* and to decide whether any interaction observed will cause a gain or loss in the therapeutic index.

This work would not have been possible without the generous cooperation and support of Professor G. E. Adams and Dr E. M. Fielden of the Radiobiology Unit, Institute of Cancer Research to P.B.R. while the latter was on study leave at the Institute.

### REFERENCES

BELLI, J. A. & PIRO, A. J. (1977) The interaction between radiation and adriamycin damage in mammalian cells. *Cancer Res.*, **37**, 1624.

BYFIELD, J. E., LYNCH, M., KULHANIAN, F. & CHAN, P. Y. M. (1977) Cellular effects of combined adriamycin and X-irradiation damage in human tumour cells. *Int. J. Cancer*, **19**, 194.

COOKE, B. C., FIELDEN, E. M., JOHNSON, M. & SMITHEN, C. E. (1976) Polyfunctional radiosensitizers: I. Effects of a nitroxyl biradical on the survival of mammalian cells *in vitro*. *Radiat. Res.*, **65**, 152.

CYSYK, R. L., SHOEMAKER, D. & ADAMSON, R. H. (1977) The pharmacologic disposition of 4'-(acridinylamino)-methanesulfon-m-anisidide in mice and rats. *Drug Metab. Dispos.*, **5**, 579.

D'ANGIO, G. J. (1962) Clinical and biologic studies of actinomycin D and roentgen irradiation. *Am. J. Roentgenol.*, **87**, 106.

DENNY, W. A., ATWELL, G. J. & CAIN, B. F. (1978) Potential antitumour agents. 26. Anionic congeners of the 9-anilinoacridines. *J. Med. Chem.*, **21**, 5.

DONALDSON, S. S., GLICK, J. M. & WILBUR, J. R. (1974) Adriamycin activating a recall phenomenon after radiation therapy. *Ann. Intern. Med.*, **81**, 407.

ELKIND, M. M., SAKAMOTO, K. & KAMPER, C. (1968) Age-dependent toxic properties of actinomycin D and X-rays in cultured Chinese hamster cells. *Cell Tissue Kinet.*, **1**, 209.

ELKIND, M. M., SUTTON-GILBERT, H., MOSES, W. B. & KAMPER, C. (1967) Sub-lethal and lethal radiation damage. *Nature*, **214**, 1088.

ELKIND, M. M. & WHITMORE, G. F. (1967) *The radiobiology of cultured mammalian cells.* New York: Gordon & Breach.

ELKIND, M. M., WHITMORE, G. F. & ALESCIO, T. (1964) Actinomycin D: Suppression of recovery in X-irradiated cells. *Science*, **143**, 1454.

GORMLEY, P. E., SETHI, V. S. & CYSYK, R. L. (1978) Interaction of 4'-(9-acridinylamino)-methanesulfon-m-anisidide with DNA and inhibition of oncornavirus reverse transcriptase and cellular nucleic acid polymerases. *Cancer Res.*, **38**, 1300.

MILLAR, B. C., FIELDEN, E. M. & MILLAR, J. L. (1978) Interpretation of survival curve data for Chinese hamster cells, line V-79, using the multi-target, multi-target with initial slope, and $\alpha$, $\beta$ equations. *Int. J. Radiat. Biol.*, **33**, 599.

PHILLIPS, T. L. & FU, K. K. (1979) The interaction of drug and radiation effects on normal tissues. *Int. J. Radiat. Oncol. Biol. Phys.*, **5**, 59.

PIRO, A. J., TAYLOR, C. C. & BELLI, J. A. (1975) Interaction between radiation and drug damage in mammalian cells. 1. Delayed expression of actinomycin D/X-ray effects in exponential and plateau phase cells. *Radiat. Res.*, **63**, 346.

POWELL, M. J. D. (1971) Recent advances in unconstrained optimization. *Math. Prog.*, **1**, 26.

ROBERTS, P. B., DENNY, W. A. & CAIN, B. F. (1979) Radiation sensitization of *E. coli* B/r by 9-anilinoacridines. *Br. J. Cancer*, **40**, 641.

ROZENCWEIG, M., VON HOFF, D. D., CYSYK, R. L. & MUGGIA, F. M. (1979) m-AMSA and PALA: Two new agents in cancer chemotherapy. *Cancer Chemother. Pharmacol.*, **3**, 135.

SALADINO, C. F. & BEN-HUR, E. (1978) Quinacrine-enhanced killing response of cultured Chinese hamster cells by X-rays. *Res. Comms. Chem. Pathol. Pharmacol.*, **22**, 629.

SINCLAIR, W. K. (1968) Cyclic X-ray responses in mammalian cells *in vitro*. *Radiat. Res.*, **33**, 620.

SHOEMAKER, D. D., LEGHA, S. S. & CYSYK, R. L. (1978) Selective localization of 4′-(9-acridinyl-amino)-methansulfon-m-anisidide in B16 melanoma. *Pharmacology*, **16**, 221.

TOBEY, R. A., DEAVEN, L. L. & OKA, M. S. (1978) Kinetic response of cultured Chinese hamster cells to treatment with 4′-(9-acridinylanimo)-methane-sulfon-m-anisidide-HCl. *J. Natl Cancer Inst.*, **60**, 1147.

WARING, M. J. (1976) DNA-binding characteristics of acridinyl-methanesulfonanilide drugs: Comparison with antitumour properties. *Eur. J. Cancer*, **12**, 995.